# Transcatheter Aortic Valve Implantation for Pure Native Aortic Regurgitation: A Comprehensive State-of-the-Art Review

**DOI:** 10.31083/RCM54189

**Published:** 2026-10-22

**Authors:** Joao Matteo Rampone, Samuele Sandrone, Sergio Capoccia, Federico Conrotto, Francesco Bruno, Ovidio De Filippo, Mario Iannaccone, Giacomo Boccuzzi, Fabrizio D’Ascenzo, Francesco Maisano, Gaetano Maria De Ferrari, Federico Giacobbe

**Affiliations:** ^1^Division of Cardiology, Cardiovascular and Thoracic Department, “Città della Salute e della Scienza” Hospital, 10126 Turin, Italy; ^2^Division of Cardiology, Department of Medical Sciences, University of Turin, 10124 Turin, Italy; ^3^Department of Cardiology, Ospedale San Giovanni Bosco, 10154 Turin, Italy; ^4^Department of Cardiac Surgery, San Raffaele University Hospital, 20132 Milan, Italy

**Keywords:** aortic regurgitation, TAVI, TAVR, dedicated-THVs, aortic valve replacement, aortic valve implantation, pure native aortic regurgitation

## Abstract

Pure native aortic regurgitation (AR) remains a challenging and frequently undertreated valvular disease, associated with substantial morbidity and mortality. Surgical aortic valve repair or replacement remains the standard of care; however, many patients are older, frail, or at prohibitive surgical risk, creating an unmet need for less invasive therapeutic options. In this context, transcatheter aortic valve implantation (TAVI) has progressively emerged as a potential alternative. However, the application of TAVI in AR has historically been limited by the absence of annular and leaflet calcification, the presence of large and often noncircular annuli, and the resulting risks of transcatheter valve embolization or migration, residual paravalvular regurgitation, and conduction disturbances. This review summarizes contemporary evidence on TAVI for pure native AR, spanning early off-label experience with first-generation self-expanding and balloon-expandable valves to newer-generation platforms and dedicated devices. Recent observational studies and multicenter registries have shown meaningful improvements in procedural success, lower rates of residual AR and second valve implantation, and acceptable short- and mid-term mortality with contemporary off-label devices. Dedicated systems, including JenaValve Trilogy, J-Valve, and others, have further enhanced anchoring and sealing performance, achieving markedly lower rates of valve migration and significant residual regurgitation. Nonetheless, permanent pacemaker implantation remains a major limitation for several platforms. In parallel, the AURORA trial introduced an anatomy-guided strategy based on multidetector computed tomography classification and dual anchoring theory, suggesting that careful anatomical selection and optimized implantation planning may improve outcomes even with non-dedicated devices. Overall, TAVI has become a viable option for selected high-risk patients with severe symptomatic pure native AR and has now been incorporated into contemporary guidelines in this setting. Future progress will likely depend on further refinement of dedicated devices, reduction of conduction-related complications, and broader integration of patient-specific anatomical and biomechanical assessment to optimize device selection and implantation strategy.

## 1. Introduction and Epidemiology

AR, or insufficiency, is a common valvular disorder, accounting for approximately 8–13% of all cases of valvular heart disease [1,2,3]. In high-income countries, degenerative changes are the leading cause of AR, whereas in low-income countries, Rheumatic Heart Disease (RHD) is most frequently responsible [4]. The prevalence of AR is higher in men and is often associated with atrioventricular conduction disturbances in more than half of cases, while the incidence and severity increase with age, peaking between 40 and 60 years [1,2,3]. Recent population-based data further emphasize the epidemiologic burden of AR, with individuals aged ≥65 years reported to have a prevalence of clinically relevant AR ranging from approximately 1.6% to 4.5%, with moderate-to-severe grades in some community cohorts exceeding the prevalence of calcific aortic stenosis (AS) [5,6]. Long-term prognosis remains poor when severe AR is left interventionally untreated and on conservative medical therapy alone, with a 10-year mortality rate reaching up to 34% and, once symptoms occur, an annual mortality rate approaching 25% in patients with advanced heart failure symptoms (New York Heart Association (NYHA) class III–IV) [7].

Over the past two decades, Transcatheter Aortic Valve Implantation (TAVI) has revolutionized the treatment of aortic valve disease. It is now recognized as a reliable, guideline-supported option for patients with severe AS, regardless of surgical risk [8,9]. Owing to its minimally invasive nature, advances in device technology, and robust evidence from randomized clinical trials, the use of TAVI has expanded well beyond patients who are inoperable or at high surgical risk. In contrast, for patients with aortic regurgitation, surgical aortic valve replacement (SAVR) remains the standard of care and confers a significant survival benefit [10,11]. Nevertheless, referral rates for surgery are low: only about one in five patients with severe aortic regurgitation and a left ventricular ejection fraction (LVEF) of 30–50% are referred for surgery, and among those with an LVEF below 30%, referrals are even less frequent [7].

Native AR differs significantly from AS in terms of pathophysiological and anatomical characteristics. From a functional standpoint, AR can be classified according to leaflet motion and root geometry, thus helping to link the mechanism of regurgitation to procedural reparability and planning [11,12,13]. Type I AR is characterized by normal cusp motion with regurgitation related to annular or root dilatation; Type II results from excessive cusp motion, such as prolapse or flail; and Type III reflects restricted cusp motion. Furthermore, AS is typically associated with substantial leaflet and annular calcification, which provides the firm support necessary for transcatheter prostheses’ anchoring. In contrast, AR often occurs in the absence of calcification; primary AR ensues from congenital malformations, degeneration, inflammation, or infection affecting the cusps, while secondary AR develops from aortic annulus dilation, root enlargement or aortopathy associated with genetic syndromes, inflammatory diseases, infection, or dissection. These unique challenges have historically rendered AR a poor candidate for TAVI, and early experiences with first-generation devices were associated with suboptimal results [14].

Despite these procedural limitations, the clinical demand for TAVI in primary AR continues to rise. Patients with severe AR are often older and have multiple comorbidities, making them poor candidates for surgical replacement. Consequently, in recent years, TAVI has emerged as an off-label option for these high-risk patients, supported by data from registries, observational studies, and meta-analyses demonstrating encouraging outcomes with newer-generation devices: enhanced radial force, repositionable systems, and valves specifically designed for AR, including those with dedicated anchoring mechanisms, have significantly improved procedural success rates compared to earlier experiences [15] (Fig. 1).

**Fig. 1.**
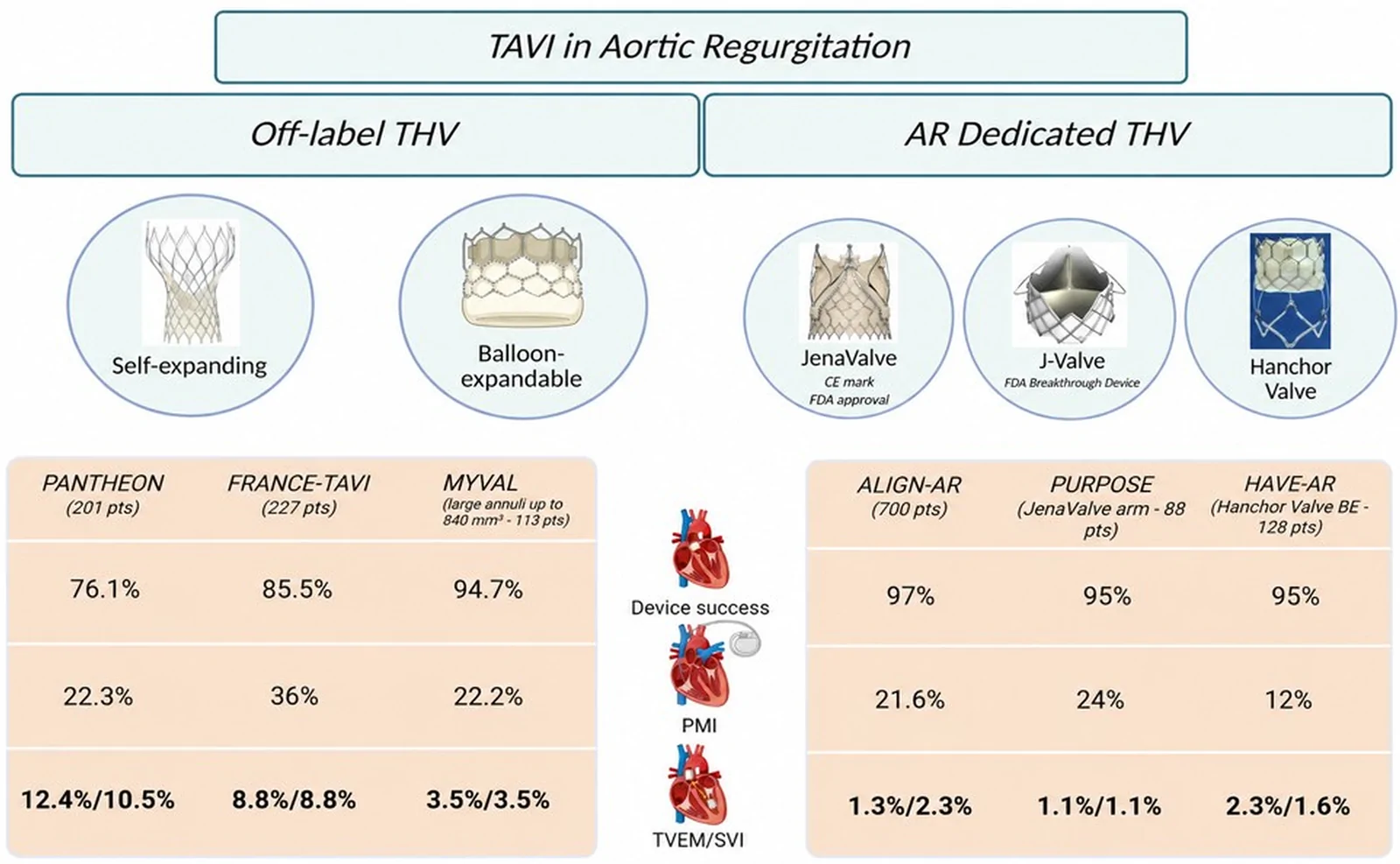
**Central illustration: contemporary transcatheter treatment strategies for pure native aortic regurgitation**. Overview of contemporary off-label SE and BE THVs and AR-dedicated devices, together with representative procedural outcomes from the PANTHEON, FRANCE-TAVI, Myval, ALIGN-AR, PURPOSE and HAVE-AR studies. The reported rows summarize device/technical success, PMI, and transcatheter valve embolization/migration or second valve implantation. Abbr.: AR, aortic regurgitation; BE, balloon-expandable; PPI, permanent pacemaker implantation; SVI, second valve implantation; THV, transcatheter heart valve; TVEM, transcatheter valve embolization or migration; CE, Conformité Européenne; FDA, United States Food and Drug Administration. Created in BioRender.

## 2. Methods

For this narrative state-of-the-art review, we searched PubMed/MEDLINE, Embase, Scopus and Google Scholar for relevant articles, from database inception through June 2026. Search terms combined “aortic regurgitation” or “aortic insufficiency” with “transcatheter aortic valve implantation”, “transcatheter aortic valve replacement”, “off-label valve”, “dedicated valve”, “surgery”, “valve repair”, “Ross procedure”, “embolization”, “migration”, “paravalvular regurgitation”, and “pacemaker”. The evidence base was assembled iteratively, with priority given to international guidelines and consensus documents, randomized and prospective studies, multicenter registries, comparative cohorts and meta-analyses. For the device-specific sections, studies were retained when they enrolled patients with pure or predominant native aortic regurgitation (AR) and reported extractable data on procedural success, complications, mortality and other clinical endpoints. Reports confined to calcific aortic stenosis, valve-in-valve procedures, mixed populations without separately reported native-AR outcomes, duplicate cohorts and isolated case reports were excluded. Because the objective was a critical narrative synthesis rather than systematic effect estimation, no formal risk-of-bias assessment or quantitative pooling was undertaken.

## 3. Treatment Indications

Native aortic valve regurgitation (NAVR) is frequently caused by primary leaflet pathology and/or dilation of the aortic annulus, which leads to diastolic retrograde flow, chronic left ventricular (LV) volume overload, compensatory LV dilatation, and eventually systolic dysfunction. Over time, this process results in symptoms such as dyspnea and reduced exercise tolerance, or progressive left ventricular remodeling with reduced ejection fraction (EF) and increased risk of heart failure and mortality, making timely valve intervention imperative [16,17,18].

Although transthoracic echocardiography (TTE) remains the cornerstone for the evaluation of AR, its severity may be underestimated in a significant proportion of patients, particularly in the presence of complex anatomical or hemodynamic conditions. Accordingly, the 2025 European Society of Cardiology/European Association for Cardio-Thoracic Surgery (ESC/EACTS) Guidelines emphasize a multimodality imaging approach, recommending cardiovascular magnetic resonance (CMR) whenever echocardiographic findings are inconclusive or discordant with the clinical presentation [8].

Assessment of AR should rely on an integrated multiparametric approach, including valve morphology, color Doppler, vena contracta, proximal isovelocity surface area (PISA), regurgitant volume and fraction, holodiastolic flow reversal in the descending aorta, LV remodeling, and aortic root dimensions. However, Doppler-derived parameters may underestimate AR severity because of eccentric jets (Coandă effect), multiple regurgitant jets, elliptical or irregular regurgitant orifices, poor acoustic windows, and parameters’ dependence on loading conditions [19]. Moreover, PISA is often unreliable in AR owing to the frequent violation of its geometric assumptions, while vena contracta measurements may be inaccurate in non-circular or multiple regurgitant orifices [20]. Although transesophageal echocardiography (TEE), particularly in three-dimensional form, provides superior anatomical definition and is invaluable for identifying the mechanism of regurgitation and surgical planning, it does not overcome these intrinsic Doppler limitations. CMR has therefore become the reference complementary imaging modality in patients with borderline or discordant findings. Using phase-contrast velocity mapping, CMR directly quantifies regurgitant volume and regurgitant fraction independently of jet morphology, providing highly reproducible measurements with excellent interobserver agreement [21]. In addition, CMR is the gold standard for assessing LV volumes, mass, and function and allows myocardial tissue characterization through late gadolinium enhancement and T1 mapping. Importantly, CMR-derived regurgitant fraction has demonstrated greater prognostic value than echocardiography, correlating more closely with symptom progression, adverse LV remodeling, and the need for aortic valve intervention. Notably, a regurgitant fraction ≥30–35% has been associated with a significantly increased risk of clinical progression, even in patients classified as having only moderate AR by echocardiography [22].

Approaching the operative side, surgical repair or replacement remains the reference standard for severe chronic AR when anatomy and operative risk are suitable. The 2025 ESC/EACTS guidelines reaffirm surgery (Class I) as the preferred intervention in symptomatic patients and in asymptomatic patients with left ventricular systolic dysfunction (LVEF <50%), progressive LV dilatation (left ventricular end-systolic diameter (LVESD) >50 mm or left ventricular end-systolic diameter index (LVESDi) >25 mm/m^2^), or in those requiring concomitant cardiac or aortic surgery, where timely intervention helps prevent irreversible myocardial remodeling and adverse events. Valve-sparing root replacement and cusp repair techniques are recommended when valve morphology and surgical expertise permit, particularly in younger patients, to preserve native tissue and avoid prosthesis-related complications [8]. In parallel, the 2020 American College of Cardiology/American Heart Association (ACC/AHA) guidelines are broadly concordant regarding Class I surgery for symptomatic severe AR but differ slightly in their thresholds for earlier intervention in asymptomatic disease. In particular, they support surgery in patients with LVEF ≤55% (Class I) and also include a Class IIb recommendation for intervention in asymptomatic patients with preserved EF who show progressive decline in LVEF to 55%–60% on serial studies or progressive LV dilatation, including left ventricular end-diastolic diameter (LVEDD) >65 mm [9].

Patient selection should be individualized by a multidisciplinary Heart Team, integrating symptom status, multimodal imaging (including echocardiography, multidetector computed tomography (CT), and CMR when needed), surgical risk scores, and life expectancy to define the optimal timing and modality of intervention [21,23,24,25].

Concomitant replacement of the ascending aorta or root is advised when diameters exceed specified thresholds at the time of valve surgery, in order to reduce future aortic events and optimize long-term outcomes [8].

When a durable repair is achievable, this approach is favored over replacement to preserve ventricular geometry; however, replacement with a bioprosthesis or mechanical valve remains indicated when repair durability is uncertain.

In the particular context of younger patients with connective tissue disorders or root enlargement phenotypes, current guidelines prioritize valve-sparing root procedures performed in experienced centers to balance long-term durability with avoidance of lifelong anticoagulation.

Importantly, emerging data suggest that traditional triggers based on symptoms, EF, and linear diameters may identify intervention too late in some asymptomatic patients [26,27,28]. In selected low-risk individuals, earlier surgery may be justified when subtle but progressive LV decompensation is documented, including LVESDi >20–22 mm/m^2^, left ventricular end-systolic diameter index (LVESVi) ≥45 mL/m^2^, or worsening myocardial mechanics such as impaired global longitudinal strain, even in the presence of apparently preserved systolic function [16,29,30].

Moreover, the Ross procedure represents an important alternative to prosthetic aortic valve replacement in this setting of carefully selected young and middle-aged adults requiring aortic valve surgery. By replacing the diseased aortic valve with the patient’s own pulmonary autograft and reconstructing the right ventricular outflow tract with a homograft, the Ross procedure provides excellent hemodynamic performance, physiological valve dynamics, and the potential for long-term durability without lifelong anticoagulation. Contemporary studies have demonstrated improved long-term survival, reduced valve-related complications, and superior quality of life compared with mechanical valve replacement in experienced centers [31,32]. However, the procedure is technically demanding and effectively converts single-valve disease into a two-valve operation, requiring careful patient selection and surgical expertise. Accordingly, current international guidelines recognize the Ross procedure as a valuable option for younger patients when performed in high-volume centers with dedicated experience, following a multidisciplinary Heart Team discussion and informed patient preference.

However, a substantial subset of patients with severe AR are elderly, frail, burdened by multiple comorbidities, or have undergone prior cardiac surgery, making them poor candidates for open surgery. In these high or prohibitive surgical risk patients, percutaneous TAVI has emerged as a potential alternative, although it poses unique technical and pathophysiological challenges compared with the more common setting of AS [33,34]. In AR, the aortic annulus is often dilated and non-calcified, and leaflet calcification, which in AS serves as an anchoring substrate, is absent or minimal. In addition, high stroke volume, dynamic aortic root geometry, and mobile leaflets may be present, all of which predispose to inadequate anchoring, migration or embolization of the transcatheter prosthesis, and significant residual paravalvular regurgitation (PVR) after the procedure, which can adversely affect both immediate and long-term hemodynamic and clinical outcomes [35].

In light of the new recommendation (Class IIb, LoE B) in the 2025 ESC Guidelines on Valvular Heart Disease, TAVI for pure native AR is now recognized as a reasonable alternative in carefully selected patients with severe symptomatic AR, high or prohibitive surgical risk, and favorable aortic annular anatomy, when performed in centers with substantial expertise and a structured Heart Team–based decision process [8]. For the time being, surgical repair remains the standard of care when feasible, particularly in younger or lower-risk patients and when a durable result is expected, whereas percutaneous treatment is reserved for selected high-risk individuals.

### 3.1 Unsuitable Anatomy and Contraindications to TAVI in NAVR

Beyond the general framework of surgical risk and Heart Team assessment, some patients may not be eligible for transcatheter approach from the outset: indeed, several anatomical and clinical features identify patients who are poor candidates for percutaneous treatment of NAVR, regardless of operative risk. Active infective endocarditis, aortic dissection, and intracardiac or valvular tumors represent absolute contraindications, since AR related to these conditions requires surgical source control and is not addressable by prosthesis-based percutaneous correction [8]. Severe aortic root or ascending aortic dilatation, annular perimeters exceeding the sizing range of currently available devices, bicuspid anatomy, and leaflet prolapse or flail each represent further well-characterized anatomical limitations to dedicated-device TAVI, accounting for a substantial proportion of screening failures in pivotal trials: these features are discussed in detail later in this review together with the corresponding device-specific sizing ranges and trial exclusion data. Finally, as already noted above, AR occurring in the context of heritable connective tissue disease (e.g., Marfan or Loeys-Dietz syndrome) is generally unsuitable for TAVI, while surgery is preferred since it addresses both the valvular and aortic components of disease and offers durable protection against future dissection or aneurysm progression; a benefit percutaneous valve replacement alone cannot provide. Taken together, anatomical suitability for TAVI in AR is considerably more restrictive than for AS: even within the high-risk population targeted by the new european guideline recommendation, only a carefully selected anatomical subset will ultimately be eligible.

### 3.2 TAVI Versus SAVR in Pure Native AR

Since percutaneous and surgical approaches currently rest on fundamentally different evidence bases, several dimensions of the comparison between SAVR and TAVI merit dedicated discussion. Despite encouraging procedural and short-to-mid-term outcomes with contemporary and dedicated devices, SAVR remains the reference standard for severe NAVR whenever anatomy and operative risk permit [8,10,11]. In the matter of durability, surgical bioprosthetic and mechanical valves have decades of follow-up establishing long-term structural performance, whereas dedicated AR-transcatheter heart valve (THV) platforms have, at most, 2-year data [36,37,38,39,40,41]. Since patients with primary AR tend to be younger than those with AS, durability is a particularly important consideration, and the absence of long-term data on leaflet degeneration or locator/frame fatigue for dedicated devices remains an important evidence gap. While second valve implantation and transcatheter valve embolization or migration (TVEM) rates have fallen to 1–2% with dedicated devices, focusing on re-intervention, valve-in-valve TAVI as a bailout for future structural deterioration has not been systematically studied in this population, and coronary re-access after supra-annular dedicated-THV implantation might also be technically demanding in younger patients likely to need future coronary intervention, as it has been reported in AS context [42]. In younger patients current guidelines and trial eligibility restrict TAVI for NAVR to high or prohibitive surgical risk; SAVR remains standard of care in younger, lower-risk patients, in whom the survival benefit of surgery is established and a lifetime valve strategy (including Ross procedure, where applicable) can be planned from the outset [8,31,32]. When AR derives from aortic root dilation or aneurysm with structurally preserved aortic leaflets, valve-sparing aortic root replacement (with aortic valve (AV) reimplantation [David] or remodeling [Yacoub]) corrects AR while preserving the native valve and avoiding anticoagulation or a bioprosthesis. In experienced centers, reoperation-free survival exceeds 70–90% at 10–15 years, with freedom from moderate-or-greater recurrent AR in the large majority of patients at 15–20 years; results are somewhat less favorable with bicuspid anatomy, though still acceptable with appropriate selection [38,39,40]. This durability, sustained over one to two decades, substantially exceeds anything yet demonstrated with transcatheter therapy. Finally, no randomized controlled trial (RCT) has yet directly compared TAVI with SAVR in NAVR; all supporting evidence for dedicated and off-label devices derives from single-arm or retrospective data using historical or performance-goal comparators. This gap is now being addressed by ARTIST Trial (Aortic Regurgitation Trial Investigating Surgery versus Trilogy™; NCT06608823, [JVT24001], https://ClinicalTrials.gov), the first RCT comparing JenaValve Trilogy against SAVR. ARTIST will enroll patients with a clinical indication for AV intervention who are not at high surgical risk and are candidates for either therapy, with a primary composite endpoint of all-cause mortality, stroke, and unplanned cardiac rehospitalization at 12 months. Its results will be critical in determining whether TAVI can be safely extended beyond today’s high-risk population and how it truly compares with surgery, including valve-sparing techniques.

## 4. Conventional Off-Label TAVI for NAVR

### 4.1 Early Experiences With First-Generation TAVI

Early off-label TAVI established procedural feasibility, but also exposed two recurrent modes of failure in non-calcified AR: inadequate anchoring, resulting in malposition or TVEM, and incomplete annular sealing, resulting in clinically relevant residual AR. In an initial CoreValve registry, device success was 74.4%, second valve implantation (SVI) was required in 18.6%, residual AR greater than mild was present in 20.9%, and mortality was 9.3% at 30 days and 21.4% at 1 year [14]. A larger landmark international registry reported device success of 74.3%, SVI in 16.6%, residual AR ≥moderate in 9.6%, new permanent pacemaker implantation (PPI) in 18.2%, and 30-day mortality of 10.9%; more importantly, its direct comparison by device generation showed that newer-generation valves increased device success from 61.3% to 81.1%, while reducing SVI from 24.4% to 12.7% and residual AR ≥moderate from 18.8% to 4.2% [34]. Sawaya et al. [33] and De Backer et al. [43] reported concordant findings: device success improved from 54% to 85% and from 47% to 82%, respectively, while SVI fell from 24% to 10% in the former and malposition from 33% to 9% in the latter; residual AR ≥moderate declined from 27% to 3% and from 31% to 4%, respectively.

Taken together, these comparisons suggest that the limitations of first-generation TAVI were not merely a reflection of early operator experience: non-repositionable frames and limited sealing capacity offered little margin for error in a large, compliant, non-calcified annulus. Failed anchoring and residual leak frequently necessitated SVI and, less commonly, urgent surgical conversion; moreover, residual AR ≥moderate was independently associated with 30-day mortality (hazard ratio (HR) 4.9 at multivariable analysis) in the Sawaya registry and with 1-year mortality in the Yoon and De Backer cohorts (HR 2.9). The early evidence therefore supported feasibility in selected inoperable patients, but not predictable performance comparable with TAVI for calcific AS [33,34,43,44,45].

### 4.2 Improvements With Newer-Generation Valves

The transition to recapturable or repositionable self-expanding frames and balloon-expandable valves with enhanced sealing skirts improved procedural control, although device sizing remained a trade-off rather than a simple pursuit of maximal oversizing. In the Yoon registry, mean perimeter oversizing was 14.8 ± 9.5% with the first-generation CoreValve and 19.9 ± 11.6% with Evolut R; among self-expanding valves, an oversizing index ≥15% was associated with less residual AR ≥moderate (4.0% vs 24.3%) [34]. Conversely, De Backer et al. [43] found that both relative undersizing and excessive oversizing were associated with malposition, particularly with the flared CoreValve/Evolut geometry, underscoring that frame-host interaction and root morphology are as important as the nominal sizing ratio. This residual uncertainty was evident in PANTHEON: despite contemporary devices, technical and device success were 83.6% and 76.1%, TVEM occurred in 12.4%, residual AR ≥moderate in 9.5%, and SVI in 10.5%. TVEM independently predicted 1-year mortality (HR 4.06) and the composite of death or heart failure (HF) hospitalization (HR 2.45), whereas postdilatation was independently associated with TVEM (odds ratio (OR) 4.06), suggesting that late radial expansion cannot reliably compensate for an unstable primary implant in the absence of calcium [15]. The S3AR study illustrated the complementary strengths and liabilities of SAPIEN 3: device success reached 94.6% and no patient had residual AR ≥moderate at 30 days, yet valve migration occurred in 10.8% and PPI in 35.1%. The investigators encouraged 15–25% oversizing, but the achieved median was approximately 12%; two embolizations were attributed to undersizing, whereas deeper left ventricular outflow tract (LVOT) implantation and greater oversizing, used to improve valve stability, were considered plausible contributors to the high PPI rate: a case of inevitable *janus bifrons* [46].

The FRANCE-TAVI and Myval experiences illustrate two contemporary but distinct approaches to the large annuli typical of NAVR. In FRANCE-TAVI, 227 patients were treated with newer-generation devices, 73% of which were ≥29 mm; technical success was 85.5%, residual AR ≥moderate was 1.2%, and SVI was required in 8.8%, at the cost of a 36.0% PPI rate. Mortality was 8.4% at 30 days, while the composite of death, HF hospitalization, or valve reintervention reached 41.6% at 1 year and 61.8% at 4 years. SVI independently predicted this endpoint (HR 1.95), supporting the interpretation that larger valves may reduce instability but do not eliminate the clinical consequences of a technically complex implant [47]. The balloon-expandable Myval platform offers standard, intermediate, and extra-large sizes (30.5 and 32 mm), permitting more granular sizing and extending nominal treatment to annular areas of 840 mm^2^ and perimeters of 100.5 mm. In 113 patients, 84.1% received an extra-large valve and mean oversizing was 17.9 ± 11.0%; technical success was 94.7%, TVEM 3.5%, residual AR ≥moderate 8.9%, PPI 15.0% at 30 days and 22.2% at 1 year, and mortality 5.3% and 9.7%, respectively. All embolizations occurred in tapered LVOT anatomies, indicating that the broader size matrix can expand eligibility for large annuli but cannot overcome an unfavorable annulus-LVOT relationship [48].

In summary, newer-generation off-label THVs have clearly improved procedural performance compared with their predecessors; however, the magnitude of benefit is heterogeneous and remains strongly anatomy-dependent. Contemporary series still report TVEM or SVI in approximately 4–12% of patients, and the PPI burden may reach 22–36% when stability is pursued through large devices, aggressive oversizing, or deeper implantation. Although 30-day mortality often fell in the single-digit range, the long-term event burden in FRANCE-TAVI demonstrated that successful correction of AR did not erase the advanced HF substrate of this population [15,46,47,48]. Accordingly, selection among off-label devices should integrate annular size, LVOT morphology, root geometry, frame behavior, sealing characteristics, and the anticipated lifetime implications of PPI or a rescue second valve, rather than rely on oversizing alone [15,34,43,47,48] (Table 1, Ref. [14,15,33,34,43,46,47,48,49,50]).

**Table 1.** **Baseline characteristics and main clinical outcomes of the principal studies evaluating off-label THVs for the treatment of pure native aortic regurgitation**.

Study	Patients	Age, years	STS-risk score (%)	EuroSCORE	Embolization/TVEM (%)	Residual AR mod/severe (%)	Second valve impl. (%)	Technical success (%)	Device success (%)	New pacemaker (%)	All-cause mortality 30 d (%)	All-cause death 1-year (%)
Roy et al., 2013 [14]	43	75.3 ± 8.8	10.2 ± 5.3	26.9 ± 17.9	-	20.9	18.6	-	74.4	16.3	9.3	21.4
Yoon et al., 2017 [34]	331	74.4 ± 12.2	6.7 ± 6.7	9.8 ± 10.7	8.5	9.6	16.6	-	74.3	18.2	10.9	24.1
NAVR (Sawaya et al., 2017) [33]	78	74.0 ± 10.0	6.7 ± 4.8	20.4 ± 11.8	-	14.0	17.0	-	72.0	18.0	14.0	-
De Backer et al., 2018 [43]	254	74.0 ± 12.0	6.6 ± 6.2	-	19.3	22.7	8.5	-	67.0	-	11.7	-
PANTHEON (Poletti et al., 2023) [15]	201	79.0 (73.0–83.0)	5.1 (4.1–6.1)	-	12.4	9.5	10.5	83.6	76.1	22.3	5.0	-
S3AR (Delhomme et al., 2023) [46]	37	81.0 (69.0–85.0)	-	4.8 (2.5–6.9)	10.8	0.0	2.7	-	94.6	35.1	8.1	16.2
Myval (Sanchez-Luna et al., 2023) [48]	113	78.4 ± 7.5	2.7 ± 1.7	3.5 ± 2.7	3.5	8.9	3.5	94.7	94.7	15.0	3.5	9.7
FRANCE-TAVI (Le Ruz et al., 2024) [47]	227	81.0 (73.5–85.0)	-	6.0 (4.0–10.9)	8.8	1.2	8.8	85.5	-	36.0	8.4	24.0
PURPOSE Trial (Off-Label THVs) (Poletti et al., 2024) [49]	168	80.0 (73.0–84.0)	3.4 (2.2–5.0)	-	15.0	10.0	11.0	81.0	73.0	22.0	-	14.4
AURORA (Yu et al., 2025) [50]	100	72.7 ± 7.2	9.1 ± 5.8	-	1.0	0.0	9.0	-	91.0	9.0	0.0	<6.0

Abbr.: AR, aortic regurgitation; EuroSCORE, European System for Cardiac Operative Risk Evaluation; STS, Society of Thoracic Surgeons; THV, transcatheter heart valve; TVEM, transcatheter valve embolization or migration. Data are reported as mean ± standard deviation, median (interquartile range), or percentages, according to the original studies.

## 5. Dedicated Transcatheter Devices for NAVR

Dedicated transcatheter valves have been engineered to address pure AR by providing secure anchoring without relying on annular calcification.

The JenaValve Trilogy system is a self-expanding porcine pericardial valve with a unique mechanism: it features three radiopaque locators that clip onto the native aortic valve leaflets, providing further and more stable anchoring in a dual manner, together with radial force, despite the absence of leaflet calcification; moreover, this mechanism enhances the seal around the THV, reducing extent and incidence of residual paravalvular leak. Early European experiences with a prior JenaValve model, requiring transapical delivery, demonstrated feasibility but were limited in scale [51,52,53]. Recently, Adam et al. [54] reported the results of transfemoral JenaValve implantation for isolated moderate-to-severe or greater, symptomatic AR in 58 patients (mean age 76.5 y, mean Society of Thoracic Surgeons (STS)-score 4.2%) from 6 centers across Germany. Device success (Valve Academic Research Consortium (VARC) 3) was 98%; no patient required a SVI or conversion to open heart surgery, and no valve embolization was reported. No moderate or severe residual AR occurred, while permanent pacemaker implantation (PPI) was required in 19.6%. At 30 days, 92% of the patients were in NYHA functional class I or II, there was no new incidence of stroke (0%) and mortality rate was 1.7%. Furthermore, the pivotal ALIGN-AR trial evaluated the transfemoral Trilogy JV in high-risk (mean Society of Thoracic Surgeons predicted risk of mortality (STS-PROM) 4.1%) symptomatic, greater-than-moderate AR patients [36]. Among 180 patients, technical success was reported at 95%, exceeding the mean success of conventional THVs in AR. At 30 days, less than 1% of patients (1/180) had AR ≥moderate, and TVEM occurred in 2.2%, while mortality was 2%. One-year all-cause mortality was 7.8%, and 6.2% among the pre-specified group who received successful valve implantation, thus significantly better than the performance goal of 25%. Notably, 24% of patients required a new PM implantation which is similar to rates reported for other off-label TAVI-AR trials, likely reflecting the effect of intentional oversizing (mean 14.3%), and deeper implantation to ensure anchoring. Indeed, later in the trial, reduction in implantation depth and less aggressive oversizing (mean 12.7% for the last third of the cohort) lead to a significant decrease in PPI rate to 14%. Interestingly, paired analyses showed evidence of left-ventricular remodelling, with a mean LV mass reduction from 323.7 g at baseline to 219.5 g, and mean LVESD decrease from 39.6 to 34.2 mm at 1 year. Updated data on continued access cohort (700 patients overall) have been recently published and showed consistent results, with negligible residual AR, a slightly reduced PPI rate of 21.6% and a 2-year mortality of 13.3% [37]. The Trilogy system received both Conformité Européenne (CE)-mark and recently United States Food and Drug Administration (FDA) approval for AR.

The J-Valve (JC Medical) is another dedicated AR valve, characterized by a self-expanding, low-profile Nitinol frame with bovine pericardial leaflets, and a valve-locating feature with three U-shaped anchor rings that capture the native leaflets conforming to the native aortic valve sinuses during the first stage of deployment. First implanted via transapical access, a transfemoral version is now available, and its two-stage deployment (claspers release and leaflet engagement, then valve release) allows secure anchoring even in large, non-calcified annuli, encompassing annular perimeters of 57–104 mm (vs 66–90 mm for the JenaValve trilogy (JVT)). Zhu et al. (2016) [55] reported a VARC device success of 94% in a cohort of 33 transapically treated patients (mean age 74.2 y; mean European System for Cardiac Operative Risk Evaluation (EuroSCORE) I 24.4%), with only one surgical conversion for embolization and one moderate residual AR (3%); 30-day all-cause mortality was 3%, while new PMI were required in only 2 patients (6%). At 6 months, no structural valve-related dysfunction or reintervention was noted, AR remained low with 87% of the patients graded as none or trivial, and all of them showed improved functional status [55]. Liu et al. (2018) [56] reported a multicenter series of 43 patients (age 73.9; mean logistic EuroSCORE I 25.5%) from 3 Chinese centers with transapical J-Valve implantation for at least moderate-to-severe symptomatic AR, with a procedural success rate of 97.7%, one conversion to SAVR due to valve embolism into the aortic arch and no SVI. One patient had moderate residual AR and died 25 days after the operation, leading to 30 day mortality of 2.3%; PPI rate was 4.7%. At 1 year, all-cause mortality was 4.7%, with stroke 2.3% and valve-related reintervention 7%, while 97% of patients had none/trace or mild AR, with improved functional status (95.1% at NYHA class I or II) [56]. More recently, a North American compassionate-use experience (Garcia et al. 2023 [57]) with the transfemoral J-Valve in 27 high surgical risk patients (median age 81y, STS score 4.3%) with severe symptomatic (96% at NYHA III–IV) AR demonstrated an overall procedural success of 81%, which improved to 100% in the last 15 cases; in fact, two cases required conversion to surgery in the early experience due to valve embolization, leading to device redesign and stricter anatomic selection (excluding native AR with prolapsing leaflets), and subsequent better outcomes. Three additional cases required a SVI; residual AR was graded none or trace in 77% at discharge, and moderate in 1 case (4%). At 30 days, mortality was 3.7%, stroke 3.7% (1/27), and new PPI rate 13%, while no patient had moderate or greater residual AR. Notably, 10 patients (38%) treated effectively with the J-Valve had aortic valve perimeters exceeding 85 mm, which was an exclusion criterion for participation in the ALIGN-AR trial of the JenaValve [57]. The J-Valve received FDA Breakthrough Device designation, while Conformité Européenne Mark approval is still pending.

A further recent addition to the field is the Hanchor Valve system (Healing Medical Technology), the first transfemoral balloon-expandable THV specifically dedicated to AR. In the multicenter single-arm HAVE AR trial, including 128 patients, the device was associated with a low rate of clinically relevant early adverse events, including new PPI in 12.0%, valve migration in only 3 cases, and 30-day mortality of 2.34%. These results appear to be related to its design, which combines a cobalt-chromium frame with a semifixed nitinol anchor element enabling automatic cusp alignment, strong leaflet grasping, and controlled implantation depth (median depth 5 mm), with a median oversizing ratio of only 5.1% and no more than mild paravalvular regurgitation at 30 days [58].

Finally, the PURPOSE study provided a unique direct comparison between dedicated and off-label devices [49]. In this multicenter registry enrolling 256 patients with severe AR and symptoms (73% NYHA III–IV) or LV dysfunction, deemed high-risk (mean STS score 3.1%) or inoperable by the local Heart Team, one-third received the JenaValve Trilogy and two-thirds received newest-gen off-label TAVI. The acute procedural results favored the dedicated JVT: technical success according to VARC-3 criteria was 98% vs 81% with off-label valves, and device success 95% vs 73%, primarily driven by significantly lower incidences of THV embolizations (1.1% vs 15%; *p* < 0.001), the need for a SVI (1.1% vs 11%; *p* = 0.004), and residual AR ≥moderate (1.1% vs 10%; *p* = 0.007), while conversion to surgery was 0% vs 3.6% respectively. The PPI rate was comparable between groups (24% vs 22% respectively); at multivariate analysis, incremental perimeter oversizing (ratio of the THV waist diameter to the aortic annular perimeter–derived diameter) of the device (OR: 1.05), BMI (OR: 1.09), and sinotubular junction diameter (OR: 1.08) were predictive of PMI. Notably, all-cause mortality and heart failure rehospitalization at 1 year was 14.4% for off-label vs 17.2% for JVT (log-rank *p* = 0.34), and using Cox regression analysis, the type of device choice was not associated with the composite endpoint.

Besides these results, successful use of both dedicated AR platforms depends on rigorous anatomical screening, since their leaflet-grasping/locator mechanisms were engineered and validated in a comparatively narrow anatomical setting relative to conventional THVs. Both systems require detailed pre-procedural planning with cardiac-gated multidetector computed tomography (MDCT) to confirm suitable vascular access, an annulus within the device’s treatment range, and an ascending aorta of adequate size and angulation to allow safe unsheathing and rotation of the locators before deployment [59].

Annulus perimeter defines the principal sizing boundary: JenaValve Trilogy (three sizes: 23, 25, 27 mm) treats perimeters of 66–90 mm, while J-Valve (five sizes: 22, 25, 28, 31, 34 mm) treats a wider 57–104 mm range. Thus, a meaningful proportion of patients with large, dilated NAVR-type annuli unsuitable for JenaValve Trilogy may still be candidates for J-Valve; as mentioned before, in the North American J-Valve series, 38% of successfully treated patients had annulus perimeters exceeding 85 mm (an ALIGN-AR exclusion criterion). Moreover, concomitant aortopathy is one of the most common reasons for anatomical exclusion. In ALIGN-AR, 47.9% of screened patients were ineligible, with anatomical factors accounting for roughly half: annulus outside sizing range (10.4%), horizontal aortic angulation precluding coaxial locator engagement (10.1%), and ascending aortic dilatation >50 mm (1.7%) [36,37]. A larger JenaValve size is in development, but significant annular or root dilatation will likely remain a limiting factor. Furthermore, leaflet prolapse or flail, while not a formal exclusion criterion, has been empirically associated with a higher embolization risk during J-Valve deployment, prompting stricter anatomical selection after early experience with device migration: thus, careful assessment of leaflet mobility and coaptation pattern should complement standard annular and aortic measurements when selecting patients for dedicated-device TAVI [57]. Finally, bicuspid anatomy represents perhaps the most consequential current limitation: since anchoring depends on symmetric engagement of three native leaflets, both devices were designed and validated exclusively in tricuspid anatomy and bicuspid AR was systematically excluded from ALIGN-AR and other pivotal dedicated-device studies; the asymmetric, often fused-cusp anatomy of bicuspid valves is poorly suited to a three-point locator mechanism, and off-label use of either device in this setting has not been systematically evaluated. Extending dedicated-device technology to bicuspid AR will likely require either off-label adaptation with individualized planning or next-generation platforms engineered for non-tricuspid anatomy, explicitly identified as a priority for future device development [59].

In summary, if not long-term outcomes, dedicated AR valves like the JenaValve and J-Valve have substantially improved procedural outcomes, achieving >95% success with minimal residual AR and low complication rates, effectively addressing the core issue of anchoring without valve calcifications in NAVR. The JenaValve’s clip mechanism and the J-Valve’s leaflet claspers both exemplify some sort of dual anchoring concept, securing the device both at the annulus and above it by interacting with native structures. PURPOSE provides the clearest comparative signal, showing marked reductions in TVEM, SVI, surgical conversion, and residual AR with JenaValve Trilogy, but no reduction in PPI and no difference in 1-year mortality or HF rehospitalization [49]. Indeed, concerns still remain: roughly 1 in 4 patients still require pacemakers with JenaValve, whereas J-Valve and Hanchor appear to have lower PPI incidence (5–13%), possibly due to supra-annular anchoring causing less LVOT pressure and limited implantation depth, with the *caveat* of limited sample, shorter-term studies; thus, longer-term follow-up are warranted, even though 1–2 year data are encouraging, with no structural failures reported [36,37,58]. Looking ahead, ongoing randomized trials comparing TAVR vs SAVR in operable AR are warranted and will be informative (ARTIST, NCT06608823). Also, expansion of device size ranges to treat very large roots, and refined techniques to reduce PMI are anticipated (Table 2, Ref. [37,49,54,55,56,57,58]).

**Table 2.** **Baseline characteristics and main clinical outcomes of the principal studies evaluating dedicated THVs for the treatment of pure native aortic regurgitation**.

Study	Patients	Age, years	STS-risk score (%)	EuroSCORE	Transfemoral access (%)	Embolization/TVEM (%)	Residual AR mod/severe (%)	Second valve impl. (%)	Technical success (%)	Device success (%)	New Pacemaker (%)	All-cause mortality 30 d (%)	All-cause death 1-year (%)
J-Valve (Zhu et al., 2016) [55]	33	74.2 ± 5.2	-	24.4 ± 5.1	0	3	3	0	-	94	6.1	3	-
JenaValve (Adam et al., 2023) [54]	58	76.5 ± 9.0	4.2 ± 4.3	6.1 ± 6.6	100	0	0	0	100	98	19.6	1.7	-
J-Valve (Liu et al., 2018) [56]	43	73.9 ± 5.7	-	25.5 ± 5.3	0	2.3	2.4	0	-	97.7	2.3	2.3	4.7
J-Valve (Garcia et al., 2023) [57]	27	81 (72–85)	4.3 (2.6–5.3)	-	78	7.4	0	7.4	81	81	13	4	12
ALIGN-AR 2025 (700 pts) (Makkar et al., 2025) [37]	700	79 (72–84)	3.8 ± 3.1	-	100	1.3	0.5	2.3	95	97	21.6	1.6	7.8
PURPOSE Trial 2024 (JenaValve) (Poletti et al., 2024) [49]	88	80.4 (71–84)	3.5 (2.1–5.5)	-	100	1.1	1.1	1.1	98	95	24	1.1	17.2
HAVE AR Trial (Pan et al., 2025) [58]	128	74 (70–78)	4.8 (4.2–6.5)	-	100	2.3	0	1.6	96	95	12	2.3	-

Abbr.: AR, aortic regurgitation; EuroSCORE, European System for Cardiac Operative Risk Evaluation; STS, Society of Thoracic Surgeons; THV, transcatheter heart valve; TVEM, transcatheter valve embolization or migration. Data are reported as mean ± standard deviation, median (interquartile range), or percentages, according to the original studies.

## 6. Lifetime Management

Finally, durability remains unresolved for every transcatheter strategy in pure native AR, albeit for different reasons. Conventional off-label THVs have now extensive long-term experience in calcific AS, but AR-specific durability has not been established under the distinct mechanical conditions of non-calcified roots, large annuli, deliberate overexpansion and oversizing, with follow-up extending to 4 years in FRANCE-TAVI [41,47]. Conversely, dedicated platforms provide AR-specific follow-up of only 1–2 years, which may be insufficient to capture structural valve deterioration, locator or frame fatigue, late gradients, coronary re-access need, or the feasibility of reintervention [37]. This limitation is particularly relevant because patients with primary AR may be younger than typical TAVI populations and lifetime management remains incompletely defined.

## 7. AURORA Trial: Anatomical Classification and Dual Anchoring Theory

A complementary strategy is to seek stability through multiple patient-specific anchoring zones along the root and ascending aorta rather than annular oversizing alone. The AURORA trial (Yu et al. [50], 2025) evaluated a multidetector CT-based classification and “dual anchoring theory” for a second-generation self-expanding VitaFlow (MicroPort) valve. The LVOT, anatomical annulus, and ascending aorta were assessed for the capacity to provide ≥10% oversizing: anatomies with at least two adequate zones (types 1–3) were treated, whereas type 4 anatomy (zero or one anchor point) was excluded. Of 187 screened patients, 100 anatomically suitable high-risk patients were enrolled. Device success was 91%, with one embolization, 9 SVIs, eight of which related to excessive implantation depth, and one surgical conversion; no procedural death occurred, and the 30-day PPI rate was 9%. Notably, all device failures happened in the first two-thirds of the cohort, showing a probable learning curve effect. At 1 year, there were no residual >moderate AR cases, with 86% graded as none or trace, functional status improved, and favorable LV reverse remodeling was observed. These results suggest that comprehensive CT matching and anatomical assessment may permit stable implantation with less reliance on aggressive oversizing and deep LVOT deployment, and this was associated with reduced incidence of TVEM and subsequent SVI compared to the literature, negligible residual AR and notably lower PM implantation rate than either off-label or dedicated devices. Nevertheless, despite introducing a promising conceptual framework, the non-randomized nature, the selected anatomical subset and single valve platform should be noted, and results be regarded as hypothesis-generating and worthy of further external, device-specific validation.

## 8. Complications

In NAVR, dilation of the aortic root and/or ascending aorta is often present, while leaflet calcification is typically minimal. In addition, the combination of increased tissue compliance, high stroke volume, and the “suction effect” caused by regurgitant flow contributes to greater procedural instability during THV deployment. As a result, moderate or severe residual AR and TVEM represent the primary complications of TAVI in this setting [15,34,43,60]. As noted, first-gen devices saw up to 10–20% embolization rates, while newer devices and techniques have lowered this rate (e.g., ~1–3% in SAPIEN 3 and Myval series). TVEM in NAVR treatment tends to happen on the ventricular side more frequently, partly due to favorable anatomies, such as a tapered LVOT, and is linked to a higher incidence of surgical conversion and in-hospital death [15,48]. Of note, postdilation seemed to be an independent predictor of TVEM in PANTHEON, due to mechanical interactions in the absence of leaflet calcifications [15]. On the contrary, some suggest rapid pacing during deployment, deep prosthesis implantation and aggressive oversizing (>15%) to reduce incidence of TVEM (and residual AR) despite the increased risk of causing conduction system damage and subsequent PM implantation [46].

Along with embolization, residual paravalvular AR also stood as a fairly common complication in early experiences, reaching an incidence of almost one third of patients using 1st gen off-label THVs, and seems to play a negative prognostic role on both all-cause mortality and HF rehospitalization at 1 year [34,43]. Occurrence of these complications has been significantly reduced by the development of new-gen THVs carrying longer stent frames, sealing skirts (BE), recapturability and optimized radial force (SE): these enhancements often led to better performance on hard outcomes, with improvement in the 1-year all-cause mortality rate [34].

Dedicated THVs have further improved procedural performance relative to these complications by markedly reducing TVEM and residual AR by means of different architectures: locators on the Trilogy JenaValve frame allow fixation of the native leaflets to the prosthesis, improving valve stability and incorporating them into the sealing mechanism that eliminates post-procedural AR (TVEM 1.3% and AR >moderate 0.5% at 30-days in the extended ALIGN-AR Trial [37]); three nitinol “U-shaped” anchor rings characterize a separated valve-locating feature on the J-Valve device, providing extra axial fixation and enhanced sealing by conforming to anatomical sinuses (0% residual AR >mild reported by Garcia et al. [57]) [36,49,55]. Thanks to the reduction in the rates of previous complications, a significant decrease in second valve implantation rate is noted through valve technology advancements, from the early experiences with 1st-gen THVs, which recorded 24–33% of SVI [34,61], to 8.8% with newer-gen THVs [47], and finally to 1–2% with dedicated THVs [37]. Interestingly, SVI was inconsistently associated with worse prognostic outcomes at multivariate analysis, leading to an increased event rate in the FRANCE-TAVI study (HR 1.95) [47]. Avoiding SVI could indeed be relevant beyond the index procedure: stacked frames may increase the risk of prosthesis–patient mismatch, complicate coronary re-access, increase the risk of coronary obstruction during future valve-in-valve treatment, and narrow subsequent lifetime-management options [62,63,64,65]. However, these potential benefits will require prospective medium- and long-term confirmation.

Finally, conduction disturbances and PPI remain the most persistent unresolved complications for all THV types, with reported rates ranging from 9% in AURORA to 21–24% with JenaValve Trilogy and 35–36% in S3AR and FRANCE-TAVI [37,46,47,49,50]. In AR, the need to generate fixation in a non-calcified annulus may require increased circumferential radial force and deeper LVOT implantation, thereby compressing the more susceptible atrioventricular conduction axis near the membranous septum. AR-specific data support this mechanism: incremental perimeter oversizing predicted PPI in PURPOSE, while larger valve size and pre-existing right bundle branch block were associated with PPI in the extended ALIGN-AR experience; conversely, the relatively low rates reported with Hanchor and AURORA coincided with controlled implantation depth and less aggressive oversizing, although cross-study comparisons remain confounded by anatomy and device selection [37,49,50,58]. In the context of TAVI in AS, a meta-analysis of observational studies further showed that progressive reduction in membranous septum length and in the difference between membranous septum length and implantation depth (ΔMSID), at preprocedural CT, increased the odds of conduction abnormality or PPI (OR 1.60 and 1.75 respectively, per 1-mm change); balloon postdilatation amplified both associations. The study-level analysis did not identify oversizing or valve type as significant effect modifiers, reinforcing that implantation depth relative to the individual membranous septum may be more informative than either variable in isolation. Preprocedural CT measurement of the membranous septum and an implantation target that preserves a positive ΔMSID may therefore reduce PPI, although an excessively high implant can conversely increase the risk of TVEM in AR anatomies; moreover, these data were derived predominantly from TAVI for AS and require validation in pure AR [66]. Finally, it should be noted that there are currently no medium-term association analyses on PM implantation impact on hard outcomes in the treatment of NAVR with TAVI; however the results obtained in AS, which could be reasonably applicable to the AR setting, have previously highlighted its negative prognostic impact [67]. Other procedure-related complications like vascular access sites, stroke and acute kidney injury (AKI) occur at rates comparable to TAVI in AS using contemporary devices.

## 9. Conclusion

In conclusion, transcatheter aortic valve implantation has emerged as a viable option for high surgical risk patients with severe symptomatic native aortic regurgitation and is now recognized by international guidelines in this specific setting. Contemporary device technology has improved procedural outcomes compared to early experiences, with dedicated devices demonstrating better hemodynamic results and reduced short-medium term complication rates relative to off-label valves, provided that future progress will depend on awaited randomized evidence, longer follow-up and results on hard clinical endpoints.

Pacemaker implantation remains a persistent challenge across all device types, though anatomically guided patient selection with optimized implantation strategies shows promise in mitigating this complication. A combined approach integrating dedicated device technology with refined anatomical assessment and individualized implantation techniques may hold the greatest potential to optimize clinical outcomes.

## References

[1] Akinseye OA, Pathak A, Ibebuogu UN (2018). Aortic Valve Regurgitation: A Comprehensive Review. Current Problems in Cardiology.

[2] Flint N, Wunderlich NC, Shmueli H, Ben-Zekry S, Siegel RJ, Beigel R (2019). Aortic Regurgitation. Current Cardiology Reports.

[3] Singh JP, Evans JC, Levy D, Larson MG, Freed LA, Fuller DL (1999). Prevalence and clinical determinants of mitral, tricuspid, and aortic regurgitation (the Framingham Heart Study). The American Journal of Cardiology.

[4] Coffey S, Roberts-Thomson R, Brown A, Carapetis J, Chen M, Enriquez-Sarano M (2021). Global epidemiology of valvular heart disease. Nature Reviews. Cardiology.

[5] Gössl M, Stanberry L, Benson G, Steele E, Garberich R, Witt D (2023). Burden of Undiagnosed Valvular Heart Disease in the Elderly in the Community: Heart of New Ulm Valve Study. JACC. Cardiovascular Imaging.

[6] d'Arcy JL, Coffey S, Loudon MA, Kennedy A, Pearson-Stuttard J, Birks J (2016). Large-scale community echocardiographic screening reveals a major burden of undiagnosed valvular heart disease in older people: the OxVALVE Population Cohort Study. European Heart Journal.

[7] Dujardin KS, Enriquez-Sarano M, Schaff HV, Bailey KR, Seward JB, Tajik AJ (1999). Mortality and morbidity of aortic regurgitation in clinical practice. A long-term follow-up study. Circulation.

[8] Praz F, Borger MA, Lanz J, Marin-Cuartas M, Abreu A, Adamo M (2025). 2025 ESC/EACTS Guidelines for the management of valvular heart disease. European Heart Journal.

[9] Otto CM, Nishimura RA, Bonow RO, Carabello BA, Erwin JP, Writing Committee Members (2021). 2020 ACC/AHA Guideline for the Management of Patients With Valvular Heart Disease: Executive Summary: A Report of the American College of Cardiology/American Heart Association Joint Committee on Clinical Practice Guidelines. Journal of the American College of Cardiology.

[10] Tarantini G, Fabris T (2020). Pure aortic valve regurgitation: SAVR is the gold standard, but TAVR is another gun. Catheterization and Cardiovascular Interventions: Official Journal of the Society for Cardiac Angiography & Interventions.

[11] Baumbach A, Patel KP, Rudolph TK, Delgado V, Treede H, Tamm AR (2024). Aortic regurgitation: from mechanisms to management. EuroIntervention: Journal of EuroPCR in Collaboration with the Working Group on Interventional Cardiology of the European Society of Cardiology.

[12] Zoghbi WA, Adams D, Bonow RO, Enriquez-Sarano M, Foster E, Grayburn PA (2017). Recommendations for Noninvasive Evaluation of Native Valvular Regurgitation: A Report from the American Society of Echocardiography Developed in Collaboration with the Society for Cardiovascular Magnetic Resonance. Journal of the American Society of Echocardiography: Official Publication of the American Society of Echocardiography.

[13] Boodhwani M, de Kerchove L, Glineur D, Poncelet A, Rubay J, Astarci P (2009). Repair-oriented classification of aortic insufficiency: impact on surgical techniques and clinical outcomes. The Journal of Thoracic and Cardiovascular Surgery.

[14] Roy DA, Schaefer U, Guetta V, Hildick-Smith D, Möllmann H, Dumonteil N (2013). Transcatheter aortic valve implantation for pure severe native aortic valve regurgitation. Journal of the American College of Cardiology.

[15] Poletti E, De Backer O, Scotti A, Costa G, Bruno F, Fiorina C (2023). Transcatheter Aortic Valve Replacement for Pure Native Aortic Valve Regurgitation: The PANTHEON International Project. JACC. Cardiovascular Interventions.

[16] Alashi A, Khullar T, Mentias A, Gillinov AM, Roselli EE, Svensson LG (2020). Long-Term Outcomes After Aortic Valve Surgery in Patients With Asymptomatic Chronic Aortic Regurgitation and Preserved LVEF: Impact of Baseline and Follow-Up Global Longitudinal Strain. JACC. Cardiovascular Imaging.

[17] Gaasch WH, Carroll JD, Levine HJ, Criscitiello MG (1983). Chronic aortic regurgitation: prognostic value of left ventricular end-systolic dimension and end-diastolic radius/thickness ratio. Journal of the American College of Cardiology.

[18] deCampos D, Teixeira R, Saleiro C, Botelho A, Gonçalve L (2020). Global longitudinal strain in chronic asymptomatic aortic regurgitation: systematic review. Echo Research and Practice.

[19] Victor K, Ring L, Tsampasian V, Oxborough D, Bhattacharyya S, Hahn RT (2025). Echocardiographic assessment of aortic regurgitation: a practical guideline from the British Society of Echocardiography. Echo Research and Practice.

[20] Biner S, Rafique A, Rafii F, Tolstrup K, Noorani O, Shiota T (2010). Reproducibility of proximal isovelocity surface area, vena contracta, and regurgitant jet area for assessment of mitral regurgitation severity. JACC. Cardiovascular Imaging.

[21] Cawley PJ, Hamilton-Craig C, Owens DS, Krieger EV, Strugnell WE, Mitsumori L (2013). Prospective comparison of valve regurgitation quantitation by cardiac magnetic resonance imaging and transthoracic echocardiography. Circulation. Cardiovascular Imaging.

[22] Harris AW, Krieger EV, Kim M, Cawley PJ, Owens DS, Hamilton-Craig C (2017). Cardiac Magnetic Resonance Imaging Versus Transthoracic Echocardiography for Prediction of Outcomes in Chronic Aortic or Mitral Regurgitation. The American Journal of Cardiology.

[23] Lancellotti P, Pibarot P, Chambers J, La Canna G, Pepi M, Dulgheru R (2022). Multi-modality imaging assessment of native valvular regurgitation: an EACVI and ESC council of valvular heart disease position paper. European Heart Journal. Cardiovascular Imaging.

[24] Ko SM, Park JH, Shin JK, Kim JS (2015). Assessment of the regurgitant orifice area in aortic regurgitation with dual-source CT: Comparison with cardiovascular magnetic resonance. Journal of Cardiovascular Computed Tomography.

[25] Myerson SG, d'Arcy J, Mohiaddin R, Greenwood JP, Karamitsos TD, Francis JM (2012). Aortic regurgitation quantification using cardiovascular magnetic resonance: association with clinical outcome. Circulation.

[26] de Meester C, Gerber BL, Vancraeynest D, Pouleur AC, Noirhomme P, Pasquet A (2019). Do Guideline-Based Indications Result in an Outcome Penalty for Patients With Severe Aortic Regurgitation?. JACC. Cardiovascular Imaging.

[27] Mentias A, Feng K, Alashi A, Rodriguez LL, Gillinov AM, Johnston DR (2016). Long-Term Outcomes in Patients With Aortic Regurgitation and Preserved Left Ventricular Ejection Fraction. Journal of the American College of Cardiology.

[28] Yang LT, Michelena HI, Scott CG, Enriquez-Sarano M, Pislaru SV, Schaff HV (2019). Outcomes in Chronic Hemodynamically Significant Aortic Regurgitation and Limitations of Current Guidelines. Journal of the American College of Cardiology.

[29] Pizarro R, Bazzino OO, Oberti PF, Falconi ML, Arias AM, Krauss JG (2011). Prospective validation of the prognostic usefulness of B-type natriuretic peptide in asymptomatic patients with chronic severe aortic regurgitation. Journal of the American College of Cardiology.

[30] Alashi A, Mentias A, Abdallah A, Feng K, Gillinov AM, Rodriguez LL (2018). Incremental Prognostic Utility of Left Ventricular Global Longitudinal Strain in Asymptomatic Patients With Significant Chronic Aortic Regurgitation and Preserved Left Ventricular Ejection Fraction. JACC. Cardiovascular Imaging.

[31] El-Hamamsy I, Toyoda N, Itagaki S, Stelzer P, Varghese R, Williams EE (2022). Propensity-Matched Comparison of the Ross Procedure and Prosthetic Aortic Valve Replacement in Adults. Journal of the American College of Cardiology.

[32] Mazine A, David TE, Stoklosa K, Chung J, Lafreniere-Roula M, Ouzounian M (2022). Improved Outcomes Following the Ross Procedure Compared With Bioprosthetic Aortic Valve Replacement. Journal of the American College of Cardiology.

[33] Sawaya FJ, Deutsch MA, Seiffert M, Yoon SH, Codner P, Wickramarachchi U (2017). Safety and Efficacy of Transcatheter Aortic Valve Replacement in the Treatment of Pure Aortic Regurgitation in Native Valves and Failing Surgical Bioprostheses: Results From an International Registry Study. JACC. Cardiovascular Interventions.

[34] Yoon SH, Schmidt T, Bleiziffer S, Schofer N, Fiorina C, Munoz-Garcia AJ (2017). Transcatheter aortic valve replacement in pure native aortic valve regurgitation. Journal of the American College of Cardiology.

[35] Yousef A, MacDonald Z, Simard T, Russo JJ, Feder J, Froeschl MV (2018). Transcatheter Aortic Valve Implantation (TAVI) for Native Aortic Valve Regurgitation - A Systematic Review. Circulation Journal: Official Journal of the Japanese Circulation Society.

[36] Vahl TP, Thourani VH, Makkar RR, Hamid N, Khalique OK, Daniels D (2024). Transcatheter aortic valve implantation in patients with high-risk symptomatic native aortic regurgitation (ALIGN-AR): a prospective, multicentre, single-arm study. Lancet (London, England).

[37] Makkar RR, Thourani VH, Vahl TP, Yadav PK, McCabe JM, George I (2025). Transcatheter aortic valve implantation with the Trilogy valve for symptomatic native aortic regurgitation (ALIGN-AR): a pivotal, multicentre, single-arm, investigational device exemption study. Lancet (London, England).

[38] Sromicki J, Van Hemelrijck M, Schmiady MO, Greutmann M, Bonassin Tempesta F, Mestres CA (2023). Twenty-five year experience with aortic valve-sparing root replacement in a single teaching center. Frontiers in Cardiovascular Medicine.

[39] David TE, David CM, Feindel CM, Manlhiot C (2017). Reimplantation of the aortic valve at 20 years. The Journal of Thoracic and Cardiovascular Surgery.

[40] Zuo Y, Tan R, Qin C (2023). Outcomes of valve-sparing aortic root replacement in patients with bicuspid aortic valve and tricuspid aortic valve: a systematic review and meta-analysis. Journal of Cardiothoracic Surgery.

[41] Thyregod HGH, Jørgensen TH, Ihlemann N, Steinbrüchel DA, Nissen H, Kjeldsen BJ (2024). Transcatheter or surgical aortic valve implantation: 10-year outcomes of the NOTION trial. European Heart Journal.

[42] Tarantini G, Nai Fovino L, Belloni F, Barbierato M, Gallo F, Vercellino M (2025). The Coronary Access After TAVI (CAvEAT) Study: A Prospective Registry of CA After TAVR. JACC. Cardiovascular Interventions.

[43] De Backer O, Pilgrim T, Simonato M, Mackensen GB, Fiorina C, Veulemanns V (2018). Usefulness of Transcatheter Aortic Valve Implantation for Treatment of Pure Native Aortic Valve Regurgitation. The American Journal of Cardiology.

[44] Leon MB, Smith CR, Mack MJ, Makkar RR, Svensson LG, Kodali SK (2016). Transcatheter or Surgical Aortic-Valve Replacement in Intermediate-Risk Patients. The New England Journal of Medicine.

[45] Adams DH, Popma JJ, Reardon MJ, Yakubov SJ, Coselli JS, Deeb GM (2014). Transcatheter aortic-valve replacement with a self-expanding prosthesis. The New England Journal of Medicine.

[46] Delhomme C, Urena M, Zouaghi O, Campelo-Parada F, Ohlmann P, Rioufol G (2023). Transcatheter aortic valve implantation using the SAPIEN 3 valve to treat aortic regurgitation: The French multicentre S3AR study. Archives of Cardiovascular Diseases.

[47] Le Ruz R, Leroux L, Lhermusier T, Cuisset T, Van Belle E, Dibie A (2024). Outcomes of transcatheter aortic valve implantation for native aortic valve regurgitation. EuroIntervention: Journal of EuroPCR in Collaboration with the Working Group on Interventional Cardiology of the European Society of Cardiology.

[48] Sanchez-Luna JP, Martín P, Dager AE, Charry PD, Beltrán JR, Sánchez-Recalde Á (2023). Clinical outcomes of TAVI with the Myval balloon-expandable valve for non-calcified aortic regurgitation. EuroIntervention: Journal of EuroPCR in Collaboration with the Working Group on Interventional Cardiology of the European Society of Cardiology.

[49] Poletti E, Adam M, Wienemann H, Sisinni A, Patel KP, Amat-Santos IJ (2024). Performance of Purpose-Built vs Off-Label Transcatheter Devices for Aortic Regurgitation: The PURPOSE Study. JACC. Cardiovascular Interventions.

[50] Yu FC, Peng XP, Yao J, Yan YF, Corona S, Fu B (2025). Transcatheter aortic valve implantation in pure aortic regurgitation: one-year outcomes of the AURORA trial. EuroIntervention: Journal of EuroPCR in Collaboration with the Working Group on Interventional Cardiology of the European Society of Cardiology.

[51] Seiffert M, Bader R, Kappert U, Rastan A, Krapf S, Bleiziffer S (2014). Initial German experience with transapical implantation of a second-generation transcatheter heart valve for the treatment of aortic regurgitation. JACC. Cardiovascular Interventions.

[52] Schlingloff F, Schäfer U, Frerker C, Schmoeckel M, Bader R (2014). Transcatheter aortic valve implantation of a second-generation valve for pure aortic regurgitation: procedural outcome, haemodynamic data and follow-up. Interactive Cardiovascular and Thoracic Surgery.

[53] Silaschi M, Conradi L, Wendler O, Schlingloff F, Kappert U, Rastan AJ (2018). The JUPITER registry: One-year outcomes of transapical aortic valve implantation using a second generation transcatheter heart valve for aortic regurgitation. Catheterization and Cardiovascular Interventions: Official Journal of the Society for Cardiac Angiography & Interventions.

[54] Adam M, Tamm AR, Wienemann H, Unbehaun A, Klein C, Arnold M (2023). Transcatheter Aortic Valve Replacement for Isolated Aortic Regurgitation Using a New Self-Expanding TAVR System. JACC. Cardiovascular Interventions.

[55] Zhu D, Wei L, Cheung A, Guo Y, Chen Y, Zhu L (2016). Treatment of Pure Aortic Regurgitation Using a Second-Generation Transcatheter Aortic Valve Implantation System. Journal of the American College of Cardiology.

[56] Liu H, Yang Y, Wang W, Zhu D, Wei L, Guo K (2018). Transapical transcatheter aortic valve replacement for aortic regurgitation with a second-generation heart valve. The Journal of Thoracic and Cardiovascular Surgery.

[57] Garcia S, Ye J, Webb J, Reardon M, Kleiman N, Goel S (2023). Transcatheter Treatment of Native Aortic Valve Regurgitation: The North American Experience With a Novel Device. JACC. Cardiovascular Interventions.

[58] Pan W, Chen S, Li W, Liu X, Chen Y, Luo J (2025). Early Results of Multicenter Trial of a Novel Balloon-Expandable Valve With Anchor for Aortic Regurgitation. JACC. Cardiovascular Interventions.

[59] Norman S, Ali N, Blackman D (2026). Transcatheter Aortic Valve Implantation for Pure Aortic Regurgitation. Journal of Clinical Medicine.

[60] Kim WK, Schäfer U, Tchetche D, Nef H, Arnold M, Avanzas P (2019). Incidence and outcome of peri-procedural transcatheter heart valve embolization and migration: the TRAVEL registry (TranscatheteR HeArt Valve EmboLization and Migration). European Heart Journal.

[61] Anwaruddin S, Desai ND, Szeto WY, Hermiller JB, Sorajja P, Kodali S (2019). Self-Expanding Valve System for Treatment of Native Aortic Regurgitation by Transcatheter Aortic Valve Implantation (from the STS/ACC TVT Registry). The American Journal of Cardiology.

[62] Ribeiro HB, Rodés-Cabau J, Blanke P, Leipsic J, Kwan Park J, Bapat V (2018). Incidence, predictors, and clinical outcomes of coronary obstruction following transcatheter aortic valve replacement for degenerative bioprosthetic surgical valves: insights from the VIVID registry. European Heart Journal.

[63] Pilgrim T, Tomii D (2023). Predicting Coronary Obstruction After TAVR: Better Safe Than Sorry. JACC. Cardiovascular Interventions.

[64] Akodad M, Sellers S, Landes U, Meier D, Tang GHL, Gada H (2022). Balloon-Expandable Valve for Treatment of Evolut Valve Failure: Implications on Neoskirt Height and Leaflet Overhang. JACC. Cardiovascular Interventions.

[65] Bruno F, Rampone JM, Islas F, Gorla R, Gallone G, Melillo F (2024). Echocardiographic and clinical features of patients developing prosthesis-patient mismatch after transcatheter aortic valve replacement: Insights from the Recovery-TAVR registry. American Heart Journal.

[66] Sá MP, Van den Eynde J, Jacquemyn X, Erten O, Rodriguez R, Goldman S (2023). Computed tomography-derived membranous septum length as predictor of conduction abnormalities and permanent pacemaker implantation after TAVI: A meta-analysis of observational studies. Catheterization and Cardiovascular Interventions: Official Journal of the Society for Cardiac Angiography & Interventions.

[67] Bruno F, Munoz Pousa I, Saia F, Vaira MP, Baldi E, Leone PP (2023). Impact of Right Ventricular Pacing in Patients With TAVR Undergoing Permanent Pacemaker Implantation. JACC. Cardiovascular Interventions.

